# Effect of ligating dogs’ arteries and veins on femoral heads

**DOI:** 10.1186/s13018-022-02993-x

**Published:** 2022-02-25

**Authors:** Zhaofa Liu, Dachang Feng, Haitao Chen, Gan Tian

**Affiliations:** 1 Department of Orthopaedics, Foshan Women and Children Hospital Affiliated to Southern Medical University, No. 11 West Renmin Rd, Chancheng District, Foshan City, 528000 Guangdong China; 2 Radiology of Orthopaedics, Foshan Women and Children Hospital Affiliated to Southern Medical University, Foshan, 528000 China

**Keywords:** Femoral head necrosis, Artery and vein, Pathology, MRI, Dogs

## Abstract

**Background:**

We separately ligated the arteries and veins of dogs to establish a canine femoral head necrosis model, then compared the differences between the outcomes of the two ligation methods on canine femoral heads.

**Methods:**

Twenty-four dogs in this experiment were randomly and evenly sorted into two groups (Group A, the arterial group; and Group B, the venous group). In dogs in Group A, the unilateral deep femoral arteries of the hips were ligated. In dogs in Group B, the unilateral deep femoral veins of the hips were ligated. Two dogs from each group were randomly selected at the 2nd, 4th, 6th, 8th, 10th, and 12th weeks postoperatively and were marked as Groups A_1_–A_6_ and B_1_–B_6_ according to the selection times. The dogs underwent X-ray (DR) and a magnetic resonance imaging (MRI) plain scan (1.5 T) on both hip joints and were then sacrificed. Bilateral femoral head specimens were soaked in formalin and then decalcified. Hematoxylin–eosin (HE) staining and histopathologic evaluation were performed on the tissue sections.

**Results:**

In dogs in Group B, abnormal pathologic changes, such as adipocytes fusing into cysts, were observed at the 4th week after establishing the model. MRI scans showed abnormal signal intensity at the 6th week, and fibrocyte regrowth was demonstrated in the necrotic area of the femoral heads at the 10th week. At the same time, indicators of tissue repair and fresh granulation tissue emerged. Changes in dogs in Group A, such as interstitial haemorrhage and oedema, were not noted in pathologic sections until 6 weeks after the model was established. MRI showed abnormal signals, such as a linear low signal intensity in the weight-bearing area of the femoral heads at the 8th week. New blood vessels emerged in the necrotic area at the 12th week, while there was no proliferation of fibrocytes and tissues.

**Conclusions:**

The development and evolution of femoral head necrosis caused by ligation of the main veins of the femoral head in dogs appeared earlier than in dogs with arterial ligation, and pathologic changes, such as necrosis and repair, were more significant in dogs in the venous group than in dogs in the other group.

## Background

Avascular necrosis of the femoral head is one of the most common diseases in orthopaedics and is associated with a number of complex factors [1]. Apoptosis of bone cells leads to a gradual collapse of subchondral bone and articular cartilage and eventually collapse of the femoral heads, which causes avascular necrosis of the femoral head [2]. With widespread attention and in-depth research, the corresponding pathologic changes in different stages of avascular necrosis of the femoral heads have been established with corresponding treatment methods [2]. In the early stage of femoral head necrosis, a head-preserving procedure is the main treatment method and aims to stop the disease process and to prevent collapse of the femoral head [2, 3]. Early head-preserving treatments for femoral head necrosis include core decompression, bone grafting with or without a vascular pedicle, and tantalum rodimplantation [2, 3]. For advanced femoral head necrosis, replacement is the preferred method to reconstruct the structure and function of hip joints [2, 3]. Since the aetiology and pathogenesis of femoral head necrosis are not fully understood, controversies regarding the choice of treatment methods and the efficacy in different stages persist [1–3]. It is now widely accepted that ischaemia is an important stage and pathologic process in the development of the disease, regardless of pathogenic factors [1–3]. This important point of view has been confirmed in animal models of femoral head necrosis, in which the arteries supplying the femoral head were ligated [4, 5]. Digital subtraction angiography (DSA) examinations of patients with femoral head necrosis in clinical practice, however, showed that blood supply imaging of the femoral heads of some patients does not reveal a manifestation of arterial ischaemia but exposes signs of venous stasis, which is often referred to as a manifestation of femoral head vein obstruction [6, 7]. We aimed to learn more about the role of femoral head venous stasis or simple venous thrombosis on femoral head necrosis. For this reason, we designed a study to ligate the main feeding arteries and veins of the femoral heads of dogs to determine the effects on the femoral heads and conducted corresponding digital X-ray scans, MRI scans, and haematoxylin–eosin (HE) staining of pathologic specimens.

## Methods

### Animals and experimental design

Thirty healthy male dogs, 14–18 months old and weighing 5–8 kg, were purchased from the Animal Experiment Center of Dalian Medical University. These animals were then housed in the Animal Room of Zhongshan Hospital Affiliated to Dalian University. The animals were fed 300 g/d standard animal feed 3 times/day and were occasionally fed vegetables. All the animals had free access to water, proper sunlight, and enough warmth to regulate their vital signs and prevent the interference of disease. After 1 week of adaptation, 24 dogs were randomly assigned to groups A_1_–A_6_ (the arterial group) or groups B_1_–B_6_ (the venous group), with 2 dogs in each group. There were six dogs that were not assigned to a group. The dogs were thoroughly disinfected before modelling began, and the animal room was cleaned and disinfected to reduce the risk of postoperative infection. The dogs underwent anteroposterior X-ray and MRI (1.5 T) of both hips to rule out femoral head necrosis before surgery.

This study was approved by the Ethical Committee of our institution (E-2014-037) and proceeded according to the National Regulations on the Administration of Laboratory Animals.

### Construction of the experimental model

**Fig. 1:**
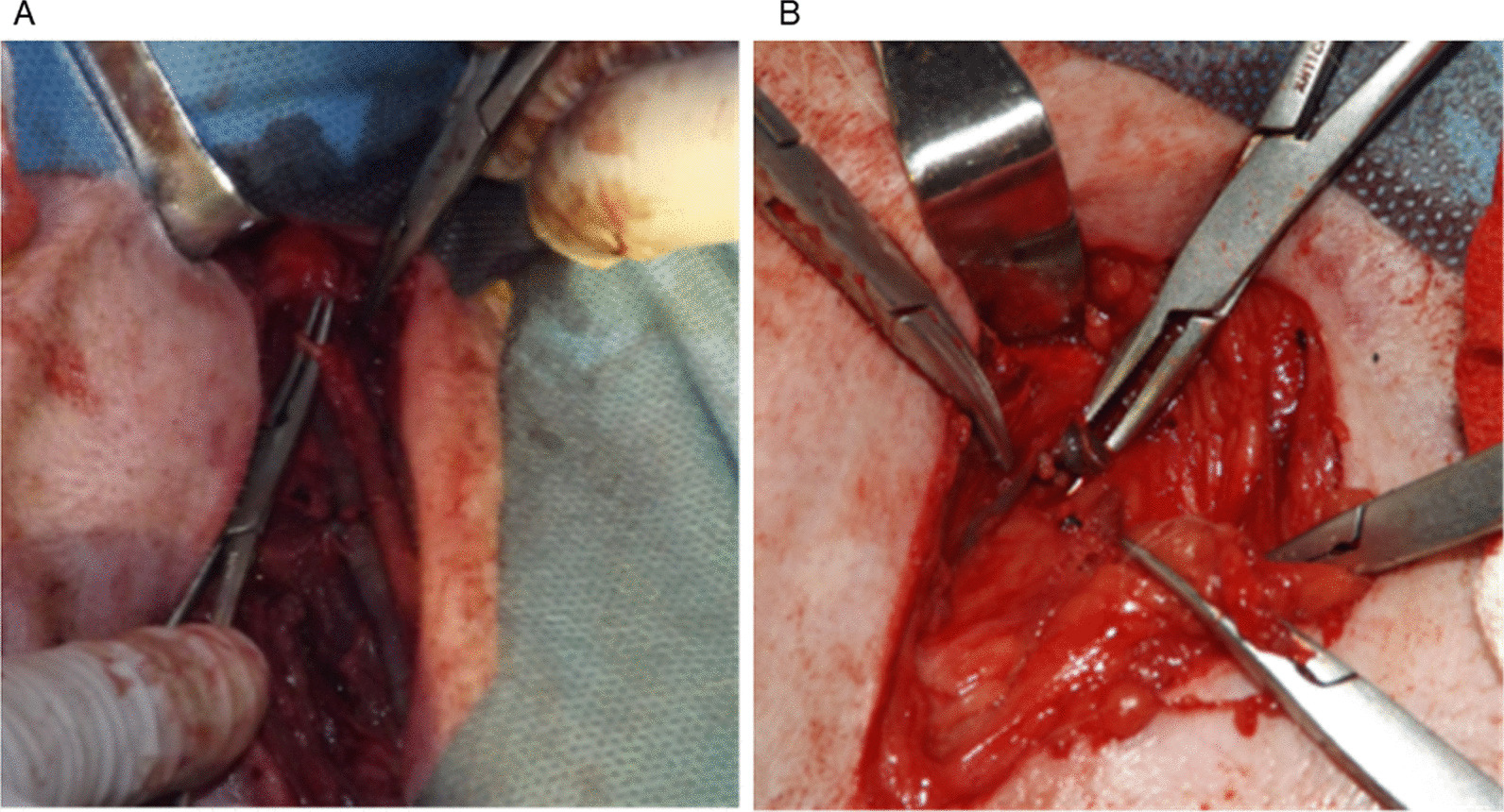
**A** The left deep femoral arteries of the dogs were ligated. **B** The left deep femoral veins of the dogs were ligated

Twenty-four experimental dogs were randomly sorted into 2 groups of 12 (Group A [deep femoral artery group] and Group B [deep femoral vein group]). The left deep femoral arteries (Group A) of the dogs were ligated with nonabsorptive surgical 4–0 silk sutures, and the left deep femoral veins (Group B) of the dogs were ligated with nonabsorptive surgical 4–0 silk sutures. After the model of ligating the canine artery and vein was established, the opposite femoral heads were designated as the control group. Anteroposterior X-ray and MRI (1.5 T) of the bilateral femoral heads of all the dogs confirmed that there was no preoperative femoral head necrosis. The left femoral heads of the dogs were selected for modelling, while the right femoral heads were used as the control group. The dogs underwent water deprivation for 6–8 h before modelling. The operating room was irradiated with ultraviolet rays for 6–8 h. Sodium pentobarbital (Bjchemmart, Beijing, China) (3% concentration) was slowly injected into the abdominal cavity of each experimental dog at a dose of 30 mg/kg. Care was taken not to inject sodium pentobarbital into the blood vessel as that would kill the experimental dog. The dogs were completely anaesthetized and placed on their backs on the operating table with their limbs secured by bandages. We covered the eyes of the dogs with black surgical towels and opened their mouths with gauze to prevent suffocation and death. If the dogs showed signs of emerging from anaesthesia during the operation, a small dose of sodium pentobarbital was injected into the dog to achieve sedation. If a dog had convulsions, we suspended the experiment immediately and prepared the dog for infusion and rescue. We used gauze to wet the hair of the dog in the surgical area, and the dog’s inguinal hair was shaved. Animal depilatory liquid was used to remove the hair to prevent burns on the skin in the surgical area. We disinfected our hands and arms with Aier iodine and then with 5% iodine and 75% alcohol in sequence for repeated deiodination until the iodine was removed. We positioned the animals, disinfected our hands and arms again, and put on sterile gowns and gloves. Through a lateral incision, the skin, subcutaneous fascia, and superficial fascia of the dogs were incised in turn. During the operation, electrocautery was used to stop the bleeding, and haemostatic forceps were used to bluntly separate the deep fascia. The gluteus medius and minimus were successively separated and excised. Lidocaine hydrochloride was injected locally around the blood vessels to expose the arteries and veins after the deep femoral blood vessels were exposed. The operated sides of the dogs were rolled outward. The left deep femoral arteries were ligated in dogs in Group A (Fig. 1A), and the left deep femoral veins were ligated in dogs in Group B (Fig. 1B). The deep femoral arteries and veins were ligated twice to achieve complete vascular ligation. After ligation, the incisions were closed in sequence according to the anatomic order, and then the dogs were returned to the Animal Experimental Center for feeding. Within 3 days postoperatively, the dogs were given intravenous infusion of gentamicin sulfate injection (80,000 Udaily) to prevent infection and intravenous infusion of glucose and normal saline at the same time to prevent shock. The vital signs of the dogs were under observation at all times, and if there were any signs of discomfort, the experimenters were notified immediately for symptomatic treatment.

### Digital X-ray (DR) and magnetic resonance imaging (MRI) examination

The dogs in Groups A_1_B_1_, A_2_B_2_, A_3_B_3_, A_4_B_4_, A_5_B_5_, and A_6_B_6_ were sacrificed by air embolism (injecting 100 ml of air intravenously) simultaneously when they underwent anteroposterior X-rays and MRIs of both hips at the 2nd, 4th, 6th, 8th, 10^th^, and 12^th^ weeks after ligation.

The magnetic field strength of magnetic resonance was 1.5 T, and conventional cross-sectional and coronal scanning were performed using phased-array head coils and fast spin-echo (FSE) sequences. The parameters were as follows: T1-weighted (TR/TE), 500/40 ms; T2, 3085/96 ms; T1 FSAT, 500/40 ms; proton density weighted, 1485/12 ms; proton lipid suppression, 1485/12 ms; matrix size, 256 × 256; FOV size, 20 cm; and layer thickness, 3.5 mm. X-ray and MRI scans were performed in a consistent order, and the differences between X-ray and MRI scans of the dogs at each time node were analysed, staged, discussed, and summarized.

### Histopathologic observation (HE staining)

Then, the bilateral femoral head specimens from the dogs were soaked in formalin for pathologic observation, cut along the coronal plane and marked separately. The specimens were decalcified with 8% hydrochloric acid tissue decalcification solution until there was no apparent hardness in cutting after they were fixed in 10% neutral formalin fixative. The specimens were repeatedly rinsed with water, embedded in paraffin and sectioned at a thickness of 5 μm; at least 5 tissue sections per femoral head were obtained. Electron microscopy was used for inspection after conventional HE staining, and photographs were obtained.

## Results

### Modelling results

Of the 24 dogs, 1 died in Group A_3,_ and 1 died in Group B_1_ after the operation. Another 2 dogs were replaced to maintain group size during the follow-up. The successfully modelled dogs did not have necrosis of the limbs on the modelling side, but the skin on the operated side had signs of a superficial infection in 1 dog in Group B2, 1 dog in Group A4, and 1 dog in Group B6. These 3 dogs were actively treated with gentamicin injection and ceftiofur sodium anti-inflammatory treatment; the infections gradually healed. Within 3 days after modelling, the dogs had poor mental states, mania, lethargy, poor appetites, and occasional fever; the dogs were treated with glucose and normal saline. The dogs’ limbs on the modelling side could not touch the ground. One week later, the dogs’ mental states and appetites gradually improved, and the claudication symptoms improved as well. The dogs walked slowly with their limbs on the modelling side.

### Dynamic observation of digital X-ray (DR)

**Fig. 2:**
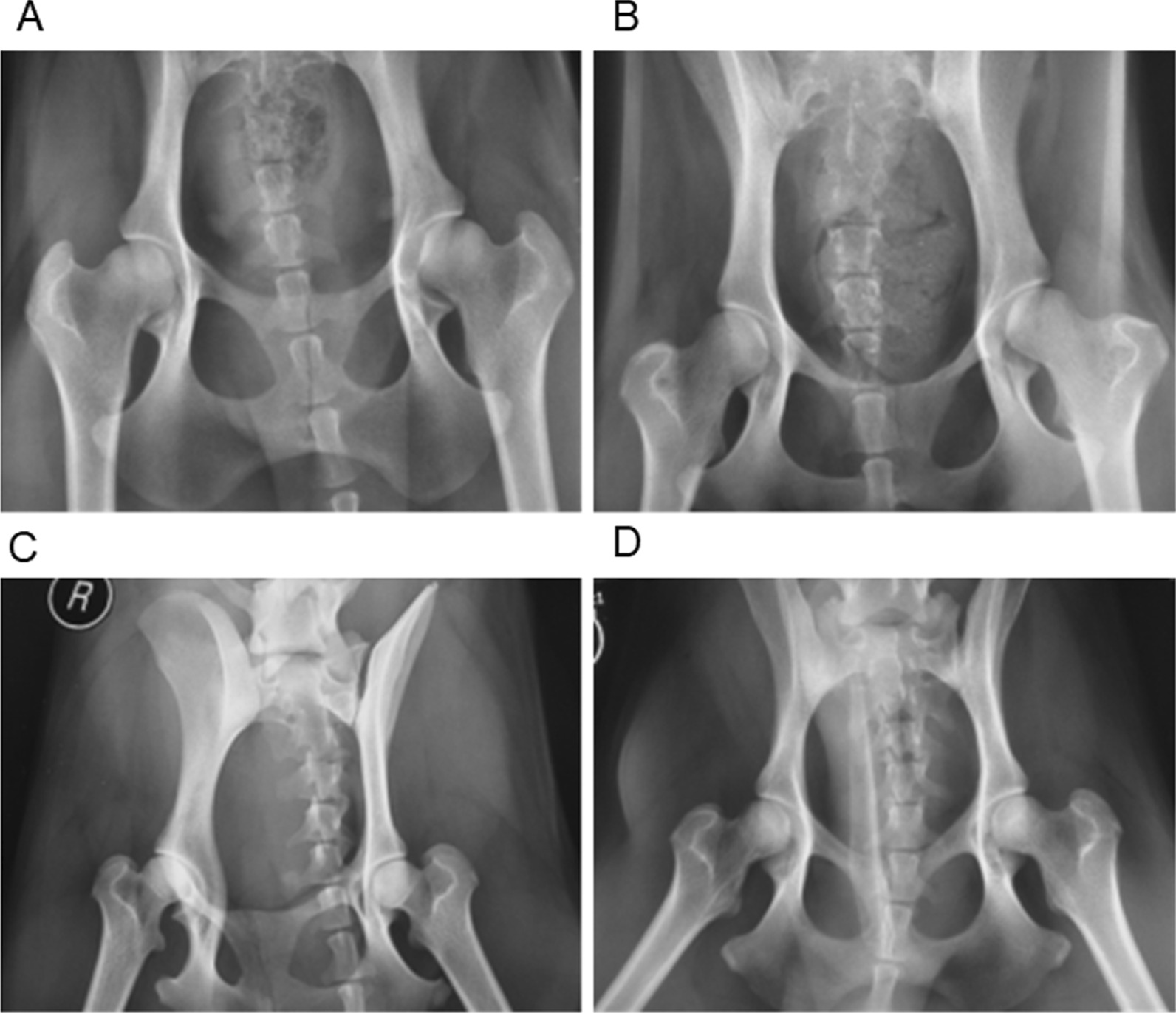
**A** The density in the femoral head was symmetric, without obvious cysts (before operation, dogs in Group A). **B** The density in the femoral head was equal, and the femoral head was smooth (before operation, dogs in Group B). **C** The femoral head was smooth, and a few cystic changes were visible (at 12 weeks, dogs in Group A). **D** The density of the femoral head was asymmetric, and there were partial sclerosis zones (at 12 weeks, dogs in Group B)

The pre- and postoperative anteroposterior X-ray images of the bilateral hip joints of the dogs were compared. The preoperative anteroposterior X-ray images of the bilateral hip joints showed a homogeneous density in the femoral heads of all dogs without cyst or sclerosis. The femoral heads were smooth without unevenness or collapse. There was no stenosis in the joint space, and the bone quality was good (Fig. 2A–D).

### Dynamic observation of MRI

**Fig. 3:**
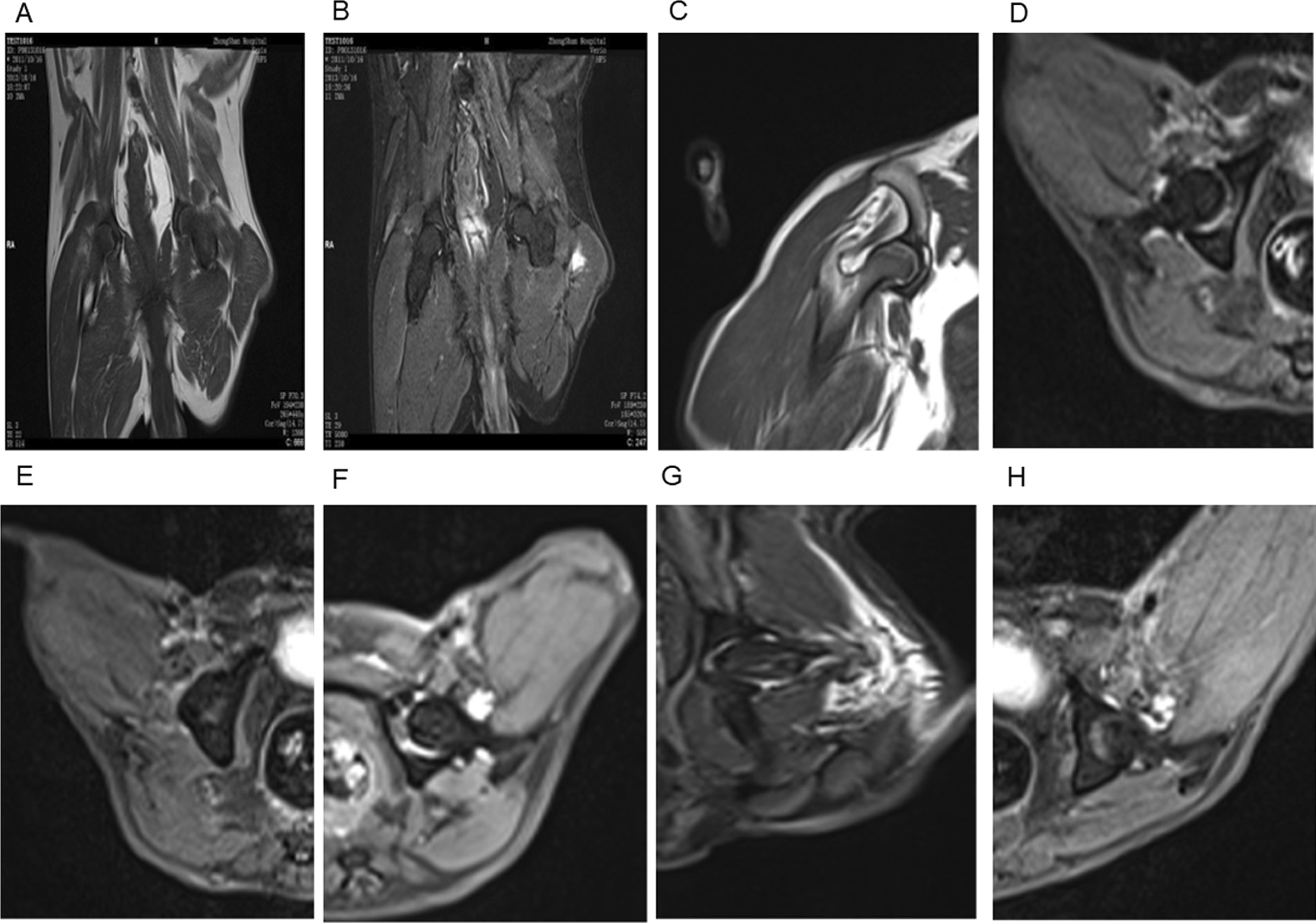
**A** A T1wMRI scan showed homogeneous low-signal intensity within the femoral head (before operation, dog in Group A). **B** A T2wMRI scan showed homogeneous low-signal intensity within the femoral head (before operation, dog in Group B). **C** A Group A_4_ MRI scan showed a stripe of T1 low-signal intensity in the weight-bearing area (at 8 weeks after operation, dog in Group A). **D** A Group A_5_ MRI scan showed elliptical T2 high-signal intensity in the weight-bearing area (at 10 weeks after operation, dog in Group A). **E** A Group A_6_ MRI scan showed a stripe of T2 high-signal intensity in the weight-bearing area (at 12 weeks after operation, dog in Group A). **F** A Group B_3_ MRI scan showed a patchy T2 slightly high -signal intensity within the femoral head (at 6 weeks after operation, dog in Group B). **G** A Group B_4_ MRI scan showed a circular T2 low-signal intensity surrounding the femoral head (at 8 weeks after operation, dog in Group B). **H** A Group B_5_ MRI scan showed a stripe of T2 high-signal intensity in the weight-bearing area (at 10 weeks after operation, dog in Group B)

T1 and T2 images of the femoral heads in normal dogs were basically the same as those of humans, showing intermediate or high signal intensity without unevenness or impurities. Preoperative MRI plain scans of the bilateral femoral heads of the dogs showed no apparent abnormal signals (Fig. 3A, B). MRI scans of the femoral heads of the dogs in Groups A_1_–A_3_ and B_1_–B_2_ (2 weeks postoperatively) showed no significant differences between the postoperative and preoperative signals. T1 images of the MRI plain scans of the bilateral femoral heads of dogs in Group A_4_ (8 weeks postoperatively) showed stripe-like, low signal intensity primarily in the weight-bearing area of the femoral heads, while a scattered dot-like, low signal intensity was primarily concentrated in the head and neck area (Fig. 3C). T2 lipid suppression images of MRI plain scans of the bilateral femoral head**s** of dogs in Group A_5_ (10 weeks postoperatively) showed an ellipsoidal shape and slightly high signal intensity in the weight-bearing area and an annular low signal intensity in the surrounding area (Fig. 3D). T2 lipid suppression images of MRI plain scans of the bilateral femoral head**s** of dogs in Group A_6_ (12 weeks postoperatively) showed stripe-like, high signal intensity in the weight-bearing area (Fig. 3E). T2 lipid suppression images of MRI plain scans of the bilateral femoral heads of dogs in Group B_3_ (6 weeks postoperatively) showed slightly high patchy signal intensity under the head, with blurred boundaries and exudate in the joint cavity (Fig. 3F). T2 femoral head images of MRI plain scans of the bilateral femoral heads of dog in Group B_4_ (8 weeks postoperatively) showed a small round sack-like area of high signal intensity surrounded by a visible circular low signal intensity (Fig. 3G). T2 lipid suppression images of MRI plain scans of the bilateral femoral head**s** of dogs in Group B_5_ (10 weeks postoperatively) showed stripe-like high signal intensity in the weight-bearing area (Fig. 3H).

### HE staining analysis

**Fig. 4:**
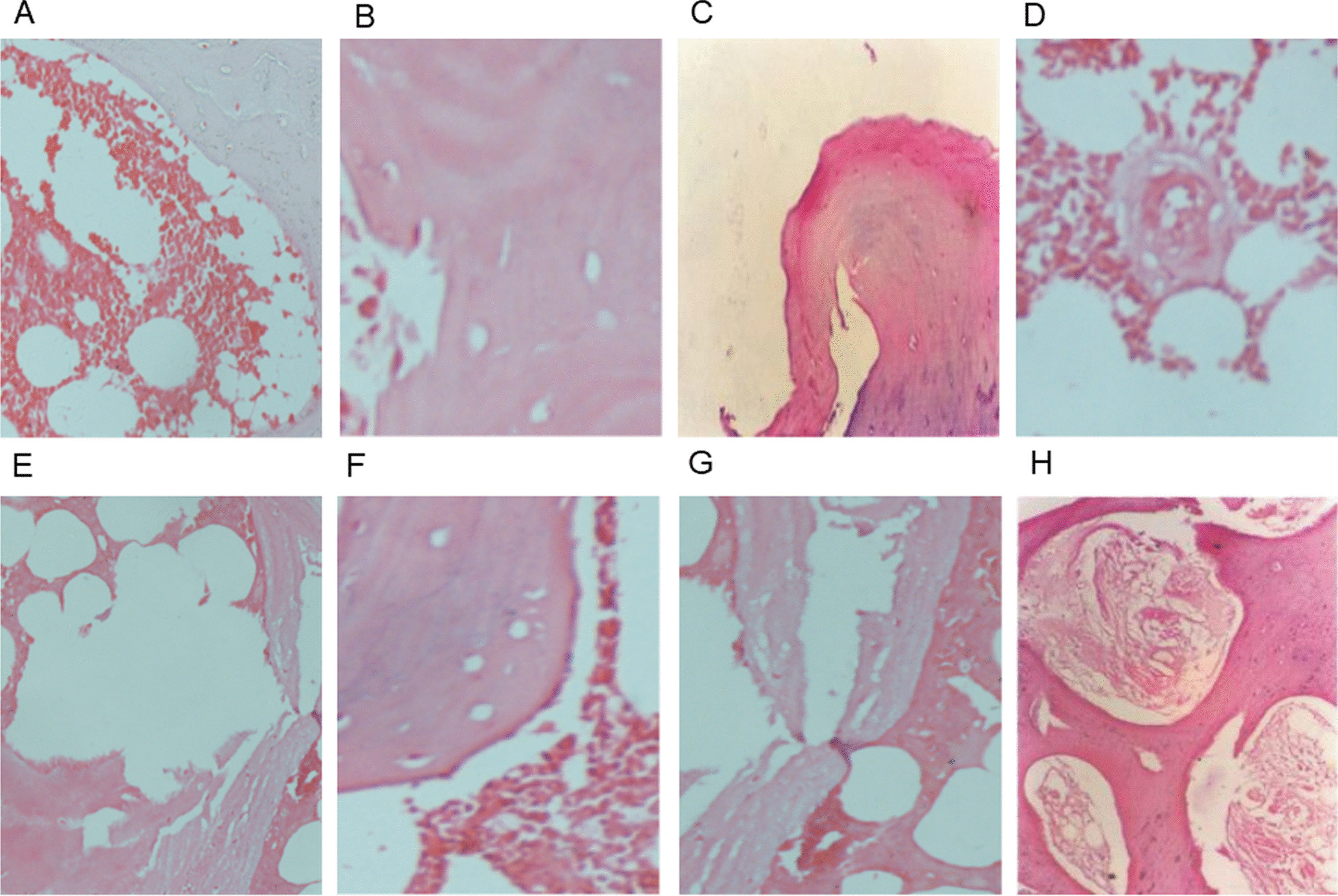
**A** Intercellular substance haemorrhage and oedema in dogs inGroup A_3_ (at 6 weeks after operation, Group A). **B** The decrease of osteocytes in bone lacuna and the fracture of the trabecular structure in dogs in Group A_4_ (at 8 weeks after operation, Group A). **C** The fracture of the trabecular bone, and the cystic degeneration of the bone tissue in dogs in Group A_5_ (at 10 weeks after operation, Group A). **D** The appearance of new blood vessels in dogs in Group A_6_ (at 12 weeks after operation, Group A). **E** The adipocytes ruptured and fused into cysts in dogs in Group B_2_ (at 4 weeks after operation, Group B). **F** The osteocytes basically disappeared in dogs in Group B_3_ (at 6 weeks after operation, Group B). **G** Fracture and structural disorder of trabecular bone in dogs in Group B_4_ (at 8 weeks after operation, Group B). **H** New granulation tissues appeared in dogs in Group B_5_ (at 10 weeks after operation, Group B)

Normal trabecular bone of the femoral head was complete with clearly visible, regular osteocytes in the trabecular bone and abundant haematopoietic cells. Pathologic changes (osteocyte oedema and intercellular substance haemorrhage in dogs in Group A; Fig. 4A) occurred at 6 weeks postoperatively. The decrease in bone lacuna osteocytes and the continuous fracture of the trabecular plate structure (Fig. 4B) occurred at 8 weeks postoperatively. The loss of osteocytes in the bone lacuna, the interruption of the continuity of the trabecular bone, and the cystic degeneration of the bone tissue (Fig. 4C) mainly appeared in the pathologic sections at 10 weeks postoperatively. New blood vessels appeared in the necrotic area of the pathologic sections at 12 weeks postoperatively, while there was no proliferation of fibrocytes and tissues (Fig. 4D). A large number of adipocytes ruptured and fused into giant adipocytes (cystic) 4 weeks postoperatively on the modelling side of dogs in Group B (Fig. 4E). The osteocytes were limited in the bone lacuna, and the trabecular plate structure remained basically the same (Fig. 4F) in the pathologic Sects. 6 weeks postoperatively. The pathologic manifestations of discontinuous and disorderly trabecular plate structure appeared in the pathologic Sects. 8 weeks postoperatively (Fig. 4G). New blood vessels emerged, and fibrocytes and granulation tissues proliferated in the necrotic area 10 weeks postoperatively (Fig. 4H).

## Discussion

The blood supply of the femoral head in humans can be categorized into two aspects: the external femoral skull artery and the internal femoral skull artery. Due to its special anatomical structure, the blood supply of the femoral head is relatively small. The medial femoral circumflex artery and the lateral femoral circumflex artery are the main blood supply sources. Injury of the medial femoral circumflex artery is the main factor leading to avascular necrosis of the femoral head [8–10]. The internal circumflex femoral artery originates from the deep femoral artery. It extends from the upper lateral side of the distal femoral neck and goes into the superior supporting artery in the joint capsule of the femoral head; alternatively, it extends from the posterior medial femoral neck and goes into the inferior supporting artery in the joint capsule of the femoral head. The lateral femoral circumflex artery originates from the deep femoral artery and has become the most important donor artery for femoral head necrosis and femoral neck fracture pedicled bone flap grafts [8–10]. Previous studies have demonstrated that the inferior gluteal artery contributes to blood flow from the arterial ring that enters the femoral head, and attention should be given to protecting the inferior gluteal artery and the medial femoral circumflex artery [9–11]. The superior retinacular vessels of the femoral head and the deep branch of the medial femoral circumflex artery are extremely important for blood supply [8–11]. Although the blood supply of the proximal femur in dogs is different from that of humans, the blood supply of the femoral head of dogs is also mainly provided by the internal and external circumflex femoral arteries, especially from the blood supply vein from the internal femoral circumflex artery. The vein flows back to the internal femoral circumflex, lateral vein, median sacral vein, and inferior gluteal vein through the supportive vein [12, 13]. Nishino et al. found that only femoral head blood flow below 20% of the control value can cause osteonecrosis in dogs, and necrotic changes are detected by MRI after ischaemia at the 4th week [14].

There are many factors leading to femoral head necrosis. The reduction in blood supply to the femoral head area is a common characteristic of femoral head necrosis [1, 2]. The histologic manifestations include interstitial haemorrhage, fibrosis, cell oedema, osteocyte ischaemia, and necrosis [1–3, 15]. In this study, the deep femoral arteries (Group A) and veins (Group B) of the dogs were ligated separately. The results of conventional DRs and MRIs (1.5 T), combined with HE staining results, showed significant differences in the pathologic changes of the femoral heads after ligating the main feeding arteries and veins of the femoral heads. First, the main pathological manifestations of the two were different. The pathological manifestations of femoral head necrosis caused by deep femoral vein ligation occurred earlier than other manifestations. In Group B, abnormal findings could be found as early as 4 weeks after ligation of the main feeding veins of the femoral heads; the fat cells in the bone marrow cavity ruptured and merged into cysts. In dogs in Group A, femoral head necrosis, evidenced by interstitial haemorrhage, oedema, etc., could be found as early as 6 weeks after ligation of the main feeding arteries. Second, the time point when the trabecular tissue started to show signs of repair was different, and the pathological manifestations were also different. In dogs in Group B, fibrocytes in the femoral head necrosis area began to regrow 10 weeks after necrosis. At the same time, there were indicators of tissue repair, such as fresh granulation tissue emerging. In dogs in Group A, manifestations of histological repair appeared 12 weeks after necrosis, namely, the formation of new blood vessels and the proliferation of fibrous tissue. Third, in the late stage of repair, the repair of necrotic bone in dogs in Group B was more obvious than that in dogs in Group A. In dogs in Group B, the formation of osteoblasts, the appearance of new bone, the proliferation and repair of cartilage tissue on the surface of necrotic bone tissue, and the proliferation of fibrous tissue with collagenization were visible. In dogs in Group A, the formation of fibrous tissue surrounding the necrotic bone was visible, and the repair was slow. In the necrosis stage, the haematopoietic cells in the bone marrow gradually decreased until they basically disappeared. In the repair stage, the haematopoietic cells in the bone marrow grew from scratch and gradually increased. Two stages showed the pathological process of the femoral head from necrosis to repair. In dogs in Group B, at the initial stage of osteonecrosis (4 weeks), fat cells increased and merged, and some of them were necrotized and dissolved. In dogs in Group A, at the initial stage of osteonecrosis (6 weeks), interstitial oedema and haemorrhage occurred. At this time, the histological changes of ischaemia and hypoxia did not cause MRI changes in intramedullary adipocytes. The changes in MRI findings did not occur until an interval of 2 weeks after the pathological changes (6 weeks in dogs in Group B, 8 weeks in dogs in Group A), which also confirms that the diagnosis of early femoral head necrosis cannot rely solely on MRI findings, and the gold standard for early detection of femoral head necrosis is pathological examination [15]. With the development of osteonecrosis (after 6 weeks in dogs in Group B and after 10 weeks in dogs in Group A), oedema, degeneration, necrosis, fragmentation and absorption of osteocytes appeared in dogs in Group A due to ischaemia and hypoxia of the femoral heads. After the reduction of osteocytes was no longer observed, the trabecular plate structure could still maintain its integrity. However, with the deepening of ischaemia and hypoxia, the trabecular plate structure gradually became disordered, broken, and necrotic, along with osteoclast phagocytosis and necrotic cellular debris of different shapes. Because of the differences in the degree of tissue damage, MRI scans showed diffusely asymmetric high and low mixed signal intensity. In dogs in Group B, microcirculation disorders, such as interstitial haemorrhage and cell oedema were visible due to blood stasis. These histologic changes were consistent with the findings previously reported in the literature [16–18]. The results for dogs in the two groups were consistent with the early phase, midphase, and late phase of blood supply changes according to the 2019 version of the Guidelines for Clinical Diagnosis and Treatment of Osteonecrosis of the Femoral Head in Adults [15]. The results in this study also demonstrated that venous stasis led to femoral head necrosis earlier than artery blood interruption. Bone cells showed signs of self-repair earlier in the necrotic process caused by venous stasis than artery blood interruption. A previous report also showed that femoral head necrosis in the early stage is often accompanied by venous stasis, and insufficient arterial blood supply is more visible in the advanced stage [6].

We hypothesize that due to the compensatory effect of the terminal vascular network, a small number of venules developed, and the blood supply of femoral heads was partially relieved. As a result, the appearance of tissue necrosis was obviously delayed. With the incomplete absorption of congestion, pathological changes in cystic degeneration gradually appeared. Because the degree of hypoxia and acidosis in dogs in the congestion group was less severe than that in dogs in the ischaemic group, the necrosis reaction and repair reaction was also expected to be milder.

There are no epidemiologic reports of femoral head necrosis anywhere in the world, but screening data of the osteonecrosis population in some countries, such as the USA, Japan, South Korea, and China, show a continuous increase [15]. Early diagnosis of osteonecrosis is crucial for treatment. MRI, X-ray imaging, CT, and bone biopsy are common methods to facilitate a diagnosis [15]. T1-weighted images (T1WIs) or “double-line signs” in T2-weighted images (T2WIs) on MRI scans are apparent signals for femoral head necrosis [19]. Many animal models have been applied in research involving femoral head necrosis for many years [20]. Dynamic observation of MRI in this study was consistent with the results in humans in previous reports [15, 19, 21, 22], which indicated that the modelling of this study has a significant reference for clinical studies.

There were some limitations in this study. First, we set the time points at 2, 4, 6, 8, 10, and 12 weeks, but the cells were still growing after 12 weeks. A long-term follow-up study may provide more evidence for further study. Second, limited by the experimental design, only MRI was used to screen the bone changes. As discussed before, MRI should not be used alone in the early stage.

## Conclusion

Overall, the occurrence, progression and repair of femoral head necrosis caused by venous stasis were significantly earlier than those caused by arterial ischaemia, and the necrosis and repair reaction of femoral heads in dogs in the venous stasis group, especially the necrosis reaction, were more obvious than that in other dogs.

## Data Availability

The datasets used during the current study are available from the corresponding author on reasonable request.

## Abbreviations

DR: Digital X-ray
MRI: Magnetic resonance imaging
DSA: Digital subtraction angiography
HE: Haematoxylin–eosin
FSE: Fast spin-echo
